# Sustainable design of high-performance multifunctional carbon electrodes by one-step laser carbonization for supercapacitors and dopamine sensors†

**DOI:** 10.1039/d4nr00588k

**Published:** 2024-04-09

**Authors:** Sanghwa Moon, Evgeny Senokos, Vanessa Trouillet, Felix F. Loeffler, Volker Strauss

**Affiliations:** a Max Planck Institute of Colloids and Interfaces Am Mühlenberg 1 14476 Potsdam Germany felix.loeffler@mpikg.mpg.de volker.strauss@mpikg.mpg.de; b Institute for Applied Materials (IAM) and Karlsruhe Nano Micro Facility (KNMFi), Karlsruhe Institute of Technology (KIT) Hermann-von-Helmholtz-Platz 1 76344 Eggenstein-Leopoldshafen Germany

## Abstract

Laser carbonization is a rapid method to produce functional carbon materials for electronic devices, but many typical carbon precursors are not sustainable and/or require extensive processing for electrochemical applications. Here, a sustainable concept to fabricate laser patterned carbon (LP-C) electrodes from biomass-derived sodium lignosulfonate, an abundant waste product from the paper industry is presented. By introducing an adhesive polymer interlayer between the sodium lignosulfonate and a graphite foil current collector, stable, abrasion-resistant LP-C electrodes can be fabricated in a single laser irradiation step. The electrode properties can be systematically tuned by controlling the laser processing parameters. The optimized LP-C electrodes demonstrate a promising performance in supercapacitors and electrochemical dopamine biosensors. They exhibit high areal capacitances of 38.9 mF cm^−2^ in 1 M H_2_SO_4_ and high energy and power densities of 4.3 μW h cm^−2^ and 16 mW cm^−2^ in 17 M NaClO_4_, showing the best performance among biomass-derived LP-C materials reported so far. After 20 000 charge/discharge cycles, they retain a high capacitance of 81%. Dopamine was linearly detected in the range of 0.1 to 20 μM with an extrapolated limit of detection of 0.5 μM (S/N = 3) and high sensitivity (13.38 μA μM^−1^ cm^−2^), demonstrating better performance than previously reported biomass-derived LP-C dopamine sensors.

## Abstract 2

Flexible and sustainable fabrication of laser patterned carbon electrodes with high performance using biowaste as a carbon source is presented.

## Graphical abstract

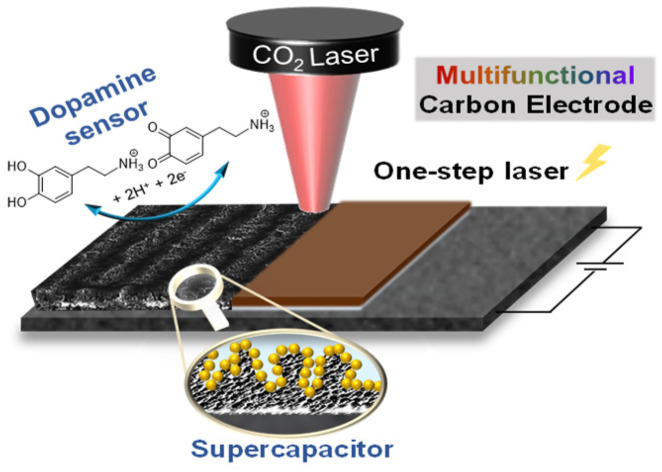

## Introduction

Electrochemical devices use electrical energy to facilitate electrochemical reactions to store as electrical energy or convert it into a readable electrical signal on electrochemical systems. They are widely used in applications ranging from energy storage or conversion to sensors, and are becoming increasingly important with respect to the electrification of many industrial, medical and environmental processes.^1–3^ Many research groups are striving to improve the performance of electrochemical systems, while there is a growing trend towards sustainable production – a concept referred to as safe and sustainable by design.^4–6^ A more sustainable design of electrochemical devices can be achieved through careful precursor selection, recyclability and reusability, efficient energy consumption, or empowering by renewable energy.

Carbon materials are widely considered sustainable electronic materials due to their abundant resource availability, lower environmental impact in the production phase compared to some materials such as metals or plastics, and inherent recyclability and reusability potential.^7–9^ Laser-carbonization, with its low energy and material requirements in laser-processing techniques, aligns well with sustainability requirements, making it a promising method for fabricating electrochemical devices.^10–12^ It enables quick production of carbon-based materials by using a laser to rapidly raise the temperature of organic materials to hundreds to thousands of degrees, causing a series of complex chemical reactions. Laser carbonization is particularly attractive because of its inherent efficiency, which results from the local application of laser-induced heat to precise, small areas of a target precursor material. This not only conserves energy and precursor material, but also enables control of the resulting material with precisely tailored properties, an important fundamental aspect in the fabrication of laser-patterned carbon (LP-C) electrodes for a variety of electrochemical applications. A notable feature of LP-C is its unique porous morphology. This porous structure is created by the evaporation of gaseous species triggered by rapid laser-induced heating and subsequent rapid cooling. This inherent high porosity is useful in applications that require a high specific surface area, such as filters, supercapacitors, sensors, and electrocatalysts. Adding to the versatility of LP-C is the ability to easily adjust the material properties and morphology. By adjusting the laser parameters and incorporating additives into the precursor material, it is possible to fine-tune important properties such as surface area, pore size distribution, and electrochemical properties. This is a promising feature for producing efficient, functional and precisely engineered carbon-based materials for electrochemical applications.

Various, mostly polymeric starting materials have been used for laser carbonization, such as polyimide (PI),^13–15^ graphene oxide (GO),^16,17^ polyacrylonitrile (PAN) fiber mats,^18,19^ or pre-carbonized molecular precursors.^20–25^ The porous nature and rich functionality of LP-C allows it to be effectively used in applications such as supercapacitors, electrocatalysis, and sensors.^14,17,18,21,23–26^ However, in many of the previous cases, heavily pre-processed or unsustainable precursor materials have been used for the laser-carbonization.

Sodium lignosulphonate (SLS) can serve as an inexpensive, abundant and sustainable carbon source. SLS is a biomass waste product from the sulfite pulping process in the paper industry and is derived from lignin, a complex polymer found in the cell walls of many plants. It is an environmentally friendly material with low toxicity and good biodegradability, making it an attractive alternative to synthetic starting materials. It has been used in various industrial processes such as animal feed, pesticides, surfactants, additives in oil drilling, stabilizers in colloidal suspensions, and as a plasticizer in concrete admixtures.^27^ Recently, SLS has also been used as a source of carbon materials in several studies related to energy materials.^28–30^

In this study, we selected SLS as a sustainable carbon precursor for LP-C and graphite foil (GF) as a current collector. We address an important issue in the fabrication process, which is the abrasion delamination of the laser-patterned carbon from the current collector. To facilitate stable adhesion of the LP-C to the conductive substrate, we introduced a polymer interlayer that acts as an adhesive between the SLS and the GF substrate. This not only provides a stable device architecture, but also allows the fabrication process to be completed in a single laser irradiation step. Our proof-of-concept strategy yields LP-C electrodes that exhibit the best performance among biomass-derived LP-C materials reported to date for both supercapacitors and dopamine sensors. It offers a sustainable electrode fabrication process due to its low energy and material consumption, fast and simple fabrication, and low-cost and renewable materials. To demonstrate the electrochemical performance of our LP-C, the electrodes were tested in symmetric supercapacitors and electrochemical sensors for selective dopamine detection.

## Results and discussion

### Fabrication of LP-C electrodes

A 17 μm graphite foil (GF) was used as a current collector, which is flexible, highly conductive, and stable in electrolytes. To achieve a stable device with strong adhesion of the LP-C to the GF, a polymer interlayer was introduced. The polymer interlayer adheres to the LP-C and the substrate, while it partially evaporates and carbonizes during the single lasing step to allow for an electrical connection between the LP-C and the current collector substrate. Absence of the polymer interlayer results in delamination of the carbon film from the substrate. Since the precursor SLS can be dissolved in hydrophilic solvents, water can be used to wash off the uncarbonized (and insufficiently carbonized) parts of the SLS after laser patterning (Fig. S1†). To act as an adhesive for SLS LP-C, the polymer interlayer must be hydrophobic to prevent delamination upon contact with water. Therefore, we chose polystyrene (PS), a global non-recyclable plastic waste, as a robust hydrophobic polymer interlayer (≈40 μm). Despite the difference in surface tension between the SLS ink film and the PS layer, SLS coating is possible when applied as a highly viscous and sticky suspension in ethylene glycol (0.9 g ml^−1^). This ensures a uniform coating regardless of the hydrophobicity of the PS.

**Fig. 1:**
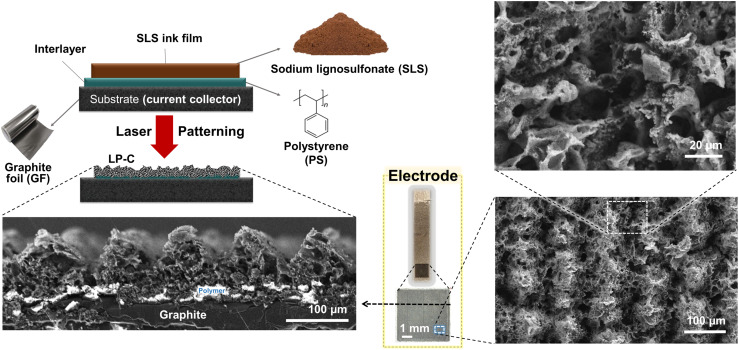
Schematic and scanning electron microscope (SEM) images showing the structure of the LP-C electrode. Precursor film for carbonization: sodium lignosulfonate (SLS); polymer interlayer: polystyrene (PS); current collector substrate: graphite foil (GF); an image in the yellow box: a real image of the LP-C electrode (PS layer in the upper part scratched to connect with the electrochemical system).

The cross-sectional scanning electron microscope (SEM) images of the washed electrodes (Fig. 1) show a typical porous morphology created by rapid laser carbonization. White (charged) areas indicate non-conductive polystyrene material that remains between the carbonized SLS film and the current collector after laser carbonization, providing stable adhesion of the LP-C to the GF substrate. The electrical connection is established by bridging zones of fully carbonized and evaporated parts of the PS layer. Due to our laser line pattern (Fig. S1†), the SEM image shows a line pattern of ridges and valleys that are visible in the cross section.

**Fig. 2:**
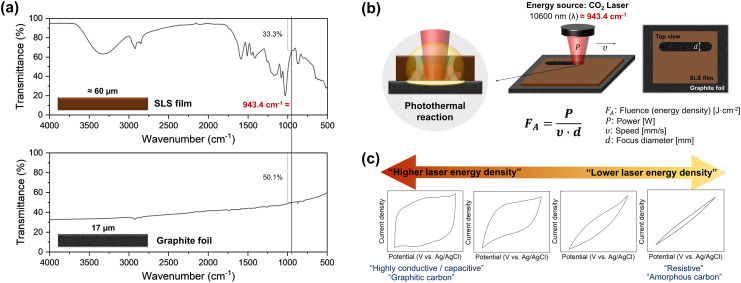
(a) Fourier transform infrared (FT-IR) spectra showing the absorbance of graphite foil and sodium lignosulfonate film at the laser wavelength (10 600 nm). (b) Schematic diagrams showing laser parameters, energy calculations, and photothermal reaction. (c) Cyclic voltammetry (CV) curves of LP-C as a function of the applied laser energy density.

To initiate the carbonization reaction with a laser, the precursor must have the property of absorbing light at the wavelength of the laser. The CO_2_ laser has a wavelength of 10.6 μm (= 943.4 cm^−1^). The FT-IR spectra show that the SLS film (≈60 μm) and the GF (17 μm) strongly absorb light at this wavelength (Fig. 2a). In the SLS, this is mainly due to vibrational modes of carbon–hydrogen (C–H) bonds, aromatic rings, hydroxyl (–OH) groups, and sulfonate (SO_3_^−^) groups. As a result, thermal energy is released and carbonization occurs. Graphite has a layered structure of stacked graphene sheets. When IR light interacts with graphite, it is absorbed and dissipated due to the high thermal conductivity of graphite. The laser energy remaining after first being used in the SLS layer is not only absorbed by the GF, but also reflected or scattered to help carbonize the SLS layer. All these phenomena together cause a photothermal reaction induced by the laser irradiation. During the rapid photothermal reaction, an oxygen deficiency occurs, which dominantly promotes the formation of C–C bonds due to the inhibited air circulation within the photothermal reaction zone, resulting in improved conductivity of carbon materials.

The main laser parameters that determine the energy density of the applied laser are power, speed, and focus diameter (Fig. 2b). The higher the laser power, the slower the scan speed, and the smaller the focus diameter, the larger is the achieved energy density.^10^ By screening various combinations of laser power and scan speed with a fixed focused diameter (≈170 μm), we found that LP-C can be tuned from highly conductive to resistive electrodes (Fig. 2c). Since electrochemical applications require highly conductive electrodes, optimal conditions for LP-C were sought for highly conductive and fully covered electrodes with LP-C. The detailed optimization data is shown in Fig. S2 and S3.† To find the optimal laser speed and power, we performed CV measurements of LP-C with varying laser parameters to determine the electrical conductivity and capacitive behavior. Slower speeds tend to produce more conductive carbon (Fig. S2†). Therefore, the optimal laser speed was set to the slowest speed of 2.4 mm s^−1^_._ However, starting at 1.2 W laser power, the electrical conductivity decreases due to material loss/ablation in the center of the laser lines caused by the high laser power (Fig. S3†). Therefore, the optimal laser power was set to 1.1 W, and a thickness of approximately 60 μm was selected through the SLS film thickness optimization experiments (Fig. S4†). The average sheet resistance of LP-C under optimized laser parameters is about 30 ohms per square.

Another advantage of this method for fabricating LP-C electrodes is that it can be applied to any flat substrate, not only GF, but also silicon wafers, flexible aluminum foil, and even glass substrates, regardless of the laser pattern (Fig. S5a–c†). In addition, when the LP-C electrode is immersed in an organic solvent, the remaining PS interlayer melts and the LP-C and GF are separated. Therefore, it is possible to separate only LP-C, and through this, the possibility of a free-standing electrode can be seen (Fig. S5d†).

### Characteristics of LP-C electrode material

**Fig. 3:**
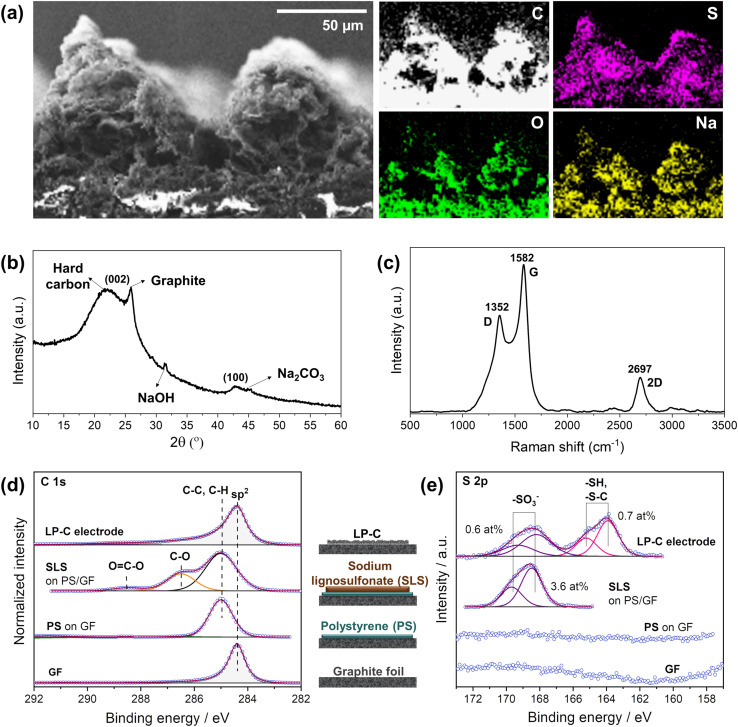
LP-C material analysis: (a) energy dispersive X-ray (EDX) mapping from scanning electron microscope (SEM), including C, S, O and Na, (b) X-ray diffraction (XRD) analysis, (c) Raman spectrum, X-ray photoelectron spectroscopy (XPS) spectra of (d) C 1s and (e) S 2p.

Energy dispersive X-ray (EDX) mapping analysis confirmed the distribution of elements in the LP-C electrode material produced by laser-carbonization with the optimized laser parameters (Fig. 3a). The cross-sectional analysis confirmed that the originally present sulfur and sodium from the SLS are uniformly distributed throughout the electrode material. In addition, the oxygen content increases slightly toward the bottom of the electrode because relatively more energy is deposited at the top due to the gradient of thermal energy generated during laser irradiation. Thus, the degree of carbonization is higher at the upper part with a higher carbon content.^23^

The LP-Cs were separated from the GF and collected as a powder for further characterization.

As shown in the SEM images in Fig. 1 and 3a, LP-C has a porous morphology. The porous nature is caused by gaseous species vaporized during the carbonization process. As the laser passes through the SLS film and the gaseous species escape, the produced LP-C cools quickly to room temperature immediately after laser irradiation, maintaining the porous structure. However, the optimal laser velocity for highly conductive LP-C is quite slow (2.4 mm s^−1^), so the pore size of the LP-C material is relatively small because the pores have more time to condense during the photothermal reaction.

N_2_ and CO_2_ gas sorption analyses were used to investigate the porous nature of LP-C (Fig. S6†). The type IV isotherm of N_2_ sorption (Fig. S6a†) reveals the presence of the mesoporous with multilayer adsorption followed by capillary condensation of nitrogen in the mesopores, manifested in a characteristic hysteresis. The specific surface area (SSA) of LP-C, estimated from the N_2_ sorption results with a density functional theory (DFT) model, is limited to 13 m^2^ g^−1^. The SSA calculated from the CO_2_ isotherm (Fig. S6c†) is much larger, corresponding to 504 m^2^ g^−1^. The CO_2_ sorption measurement is performed at high temperature (273 K), which facilitates the diffusion of gas molecules into narrower pores. This suggests that the contribution of mesopores to the total surface area of the material is negligible. Furthermore, non-local density functional theory (NLDFT) analysis confirms that most of the pores accessible to CO_2_ are <1 nm (Fig. S6d†). The total pore volume obtained from CO_2_ and N_2_ sorption is 0.21 cc g^−1^ and 0.05 cc g^−1^, respectively, further confirming major contribution of micropores to the material. Nevertheless, the collected bulk material should not be considered homogeneous. Previous studies have shown a clear gradient of different material “phases” across the LP-C film due to the energy gradient of the laser carbonization along the *z*-axis.^31^ This should be taken into account when interpreting the bulk properties.

According to the XRD spectrum, the LP-C is composed of ordered graphitic carbon and disordered hard carbon, which can be seen from the separated (002) peaks (Fig. 3b). This is also a phenomenon caused by the vertical energy gradient of the laser energy, so that the SLS is transformed into a different type of carbon along the *z*-axis. In addition, the sodium component naturally present in SLS exists in two main forms, one is NaOH and the other is Na_2_CO_3_ in lesser amounts.^32^ Since they are formed at different temperature ranges, this also further confirms the vertical energy gradient of the laser.

The Raman spectrum (Fig. 3c) shows that the LP-C has typical turbostratic carbon properties. The D band at ∼1352 cm^−1^ represents disordered carbon due to out-of-plane vibrations attributed to the presence of sp^3^ defects in graphitic-like carbon layers resulting from oxygen-containing functional groups and sulfur-doping. The G band at ∼1582 cm^−1^ is a result of in-plane vibrations of sp^2^ bonded carbon, representing ordered carbon in a graphitic structure. The degree of order or disorder in LP-C can be expressed by the ratio of the intensities of the D and G bands (*I*_D_/*I*_G_), which is 0.66, indicating few structural defects. The broad 2D peak at ∼2695 cm^−1^ denotes scattering by second-order zone boundary phonons and indicates a multilayer graphene state of LP-C.

X-ray photoelectron spectroscopy (XPS) analysis was performed to identify the elements present in the electrode material and their oxidation state. In order to accurately distinguish SLS before and after carbonization, we analyzed each step of the electrode fabrication process (GF, PS, SLS, and LP-C). As expected, GF shows an intense C 1s peak at 284.4 eV, corresponding to the graphitic sp^2^ carbon (Fig. 3d bottom). The deposition of PS on the GF is supported by the observed shift of the C 1s to 285.0 eV, together with a symmetrical shape and broadening of the previous peak. The next C 1s spectrum above shows a clear peak at 286.5 eV corresponding to C–O species. Meanwhile, an S 2p doublet with S 2p_3/2_ is observed at 168.4 eV (typical for oxidized sulfur species, *e.g.* sulfonate, Fig. 3e). All this provides evidence for the SLS coating on the PS. After laser irradiation, a clear graphitic C 1s peak can be detected for LP-C, proving the carbonization of the SLS layer. Furthermore, the amount of sulfur corresponding to the sulfonate species in the layer decreases significantly from 3.6 at% to 0.6 at%. Another S 2p doublet with S 2p_3/2_ at 163.9 eV is present at 0.7 at%, which is related to C–S bonds (Fig. 3e). Therefore, it is likely that sulfur is bound to the carbon lattice of the LP-C.

## Electrochemical applications

### Supercapacitors

**Fig. 4:**
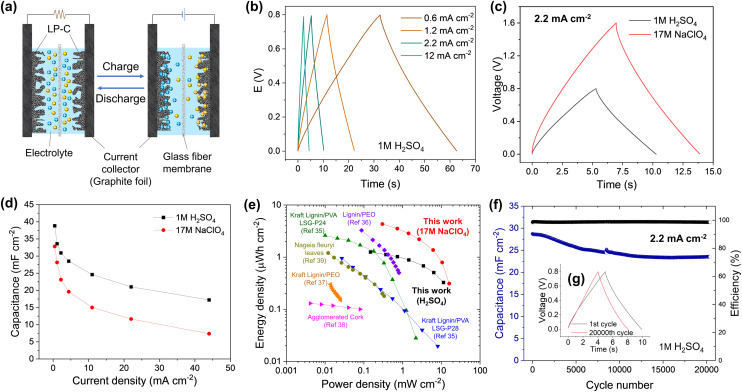
Supercapacitor performance of LP-C electrodes using a symmetric cell: (a) schematic image of the EDLC charge–discharge process; (b) Galvanostatic charge and discharge (GCD) profiles in 1 M H_2_SO_4_ at different current densities and (c) at a current density of 2.2 mA cm^−2^ for different electrolytes; (d) specific areal capacitance as a function of current density; (e) Ragone plot showing the comparison of the energy storage performance of the LP-C with the laser patterned supercapacitors using biomass-derived materials; (f) long-term cyclability at a current density of 2.2 mA cm^−2^ showing the retention of specific areal capacitance and coulombic efficiency.

To investigate the electrochemical performance of the LP-C, the electrode was characterized in a three-electrode cell system with 1M H_2_SO_4_ electrolyte. The characteristics of a supercapacitor electrode were confirmed by the rectangular-like shape of the cyclic voltammetry (CV) curves, indicating a dominant electrical double layer capacitor (EDLC) behavior related to the adsorption of the ions on the carbon surface (Fig. S7a and b†).^33^ This is the basis for charging/discharging of an EDLC device (Fig. 4a).^34^ We also compared the CV curves of bare GF and confirmed that GF does not contribute to the capacitive behavior of the LP-C electrode. SEM images of GF (Fig. S8†) show that GF has a small surface area with an almost flat surface without pores. The rectangular-like shape of the CV curves is maintained even at very high scan rates of 1 V s^−1^, demonstrating the good rate capability and high electrical conductivity of our LP-C electrodes (Fig. S7c†). This is further supported by the low equivalent series resistance (ESR) measured by impedance spectroscopy (Fig. S7d†).

To evaluate the performance of LP-C as an electrode in a supercapacitor device, a symmetrical two electrode cell was tested in aqueous 1 M H_2_SO_4_ and 17 M NaClO_4_ water-in-salt (WIS) electrolytes.

Galvanostatic intermittent titration technique (GITT) is used to characterize the electrochemical behavior of supercapacitor electrodes, providing insight into diffusion coefficients, charge transfer kinetics, and electrode/electrolyte interactions (Fig. S9†). The *D*_GITT_ values measured at different potentials are in the range of 2 × 10^−6^–18 × 10^−6^ cm^2^ s^−1^. Such high *D*_GITT_ values indicate fast diffusion of the ions through the porous of carbon network. This can be related to the large open meso- and microporosity of LP-C as well as a unique morphology of the laser-patterned carbon material providing high interface area between the electrode and the electrolyte.

Galvanostatic charge–discharge (GCD) curves in 1 M H_2_SO_4_ (Fig. 4b) appeared as nearly symmetric triangles, indicating highly reversible electrical double layer charge storage, which is reflected in high coulombic efficiency. The low ohmic drop further confirms the excellent electrical conductivity of LP-C. The voltage window limited by the water splitting reaction can be extended with superconcentrated aqueous electrolytes, namely “water-in-salt” (WIS) electrolytes. Here, the 17 M NaClO_4_ WIS electrolyte was employed achieving 1.6 V of the electrochemical window in the full cell without compromising the capacitive performance and coulombic efficiency of the electrode material (Fig. 4c). The maximum areal capacitance of 38.8 mF cm^−2^ and 32.8 mF cm^−2^ was achieved in 1 M H_2_SO_4_ and 17 M NaClO_4_, respectively, at 0.44 mA cm^−2^ (Fig. 4d). These values correspond to the highest areal capacitances reported to date for laser carbonized electrodes produced from sustainable (biomass-derived) precursors. At high currents approaching 44 mA cm^−2^, the material still exhibits 17.2 and 7.4 mF cm^−2^ in 1 M H_2_SO_4_ and 17 M NaClO_4_, respectively, indicating the excellent current-rate capability of the LP-C material. The lower capacitance in 17 M NaClO_4_ can be attributed to the lower ionic conductivity inherent to WIS electrolytes. The high ion concentration can inhibit the effective penetration of ions into some pores in the LP-C, limiting the full utilization of the active surface area and reducing the capacitance.

The Ragone plot shows a trade-off between the energy and power densities of LP-C compared to the reported sustainable (biomass-derived) LP-C supercapacitors (Fig. 4e). The LP-C-based device delivered large areal energy and power densities of 1.3 μW h cm^−2^ and 11.4 mW cm^−2^, respectively, in 1 M H_2_SO_4_. The extended voltage window in 17 M NaClO_4_ further increases device performance, achieving 4.3 μW h cm^−2^ of the specific energy and 16 mW cm^−2^ of the specific power. These values are superior to those of previously reported laser-carbonized electrodes produced from sustainable sources. It should be noted that the energy and power values were estimated from the GCD results by integrating the discharge profiles. This approach considers the shape of the GCD curves and the contribution of the ohmic drop, which is often neglected in most of the publications listed in Table S1,† using a common equation for the energy density:^35–39^

1 *E* = *CV*^2^/2

For a clearer comparison, the energy and power of LP-C were also calculated using eqn (1) and put in brackets, showing an even larger gap with the literature. It should be mentioned that most studies employ gel electrolytes (*e.g.* H_2_SO_4_/PVA), so ion diffusion might be more inhibited compared to liquid electrolytes. However, this also requires low current densities (from 0.005 to 2 mA cm^−2^) to account for larger resistances, enabling access to larger values of capacitance, energy and power densities. In this study, high surface area LP-C electrodes with a predominantly microporous nature were assembled in a conventional symmetric EDLC device. The traditional supercapacitor configuration allows employing liquid aqueous electrolytes translated to lower internal resistance and higher current densities (from 0.44 to 44 mA cm^−2^) required for high power applications. Furthermore, the LP-C film can be detached from the substrate by simply washing the PS polymer layer with some organic solvents. The free-standing LP-C electrode is sufficiently robust to maintain its integrity after drying, and it can potentially be used to fabricate flexible supercapacitor devices. The gravimetric density of the free-standing LP-C electrodes was ∼0.8 mg cm^−2^ (average of 14 LP-C electrodes). For comparison, the gravimetric performance of the supercapacitor device (normalized by the total weight of two LP-C electrodes) was also estimated (Fig. S11†).

The capacitive performance of the LP-C electrode is stable over the long-term cycling (Fig. 4f & Fig. S10b†). The LP-C-based EDLC device maintains a high coulombic efficiency of >98% and retains 81% of its initial capacitance in 1 M H_2_SO_4_ over 20 000 GCD cycles at 2.2 mA cm^−2^. Comparison of the GCD profiles shows negligible changes in electrochemical behavior after 20 000 GCD cycles (Fig. 4g). The results indicate that the fabricated symmetric LP-C supercapacitor devices have excellent performance and electrochemical stability due to the highly conductive and robust LP-C electrodes.

### Electrochemical dopamine (DA) sensor

The same LP-C electrode can also be used as an electrochemical sensor platform. In particular, it has effective selectivity for dopamine (DA) in a redox reaction over interferences from ascorbic acid (AA) and uric acid (UA) (Fig. S12a†).

The neurotransmitter dopamine plays a central role in regulating mood, movement, cognition, and the pleasure-reward system in the brain. Irregular levels of dopamine have been linked to a variety of conditions, including Parkinson's disease, schizophrenia, depression, and attention deficit hyperactivity disorder (ADHD).^40,41^ Electrochemical detection is an excellent approach to dopamine detection because of its simplicity, sensitivity, and ability to provide real-time results. In electrochemical measurements, the intrinsic redox response of dopamine results in measurable electrical signals that can quantify dopamine concentrations. In addition to high sensitivity and selectivity, this technology can be miniaturized, making it suitable for portable and point-of-care devices.

**Fig. 5:**
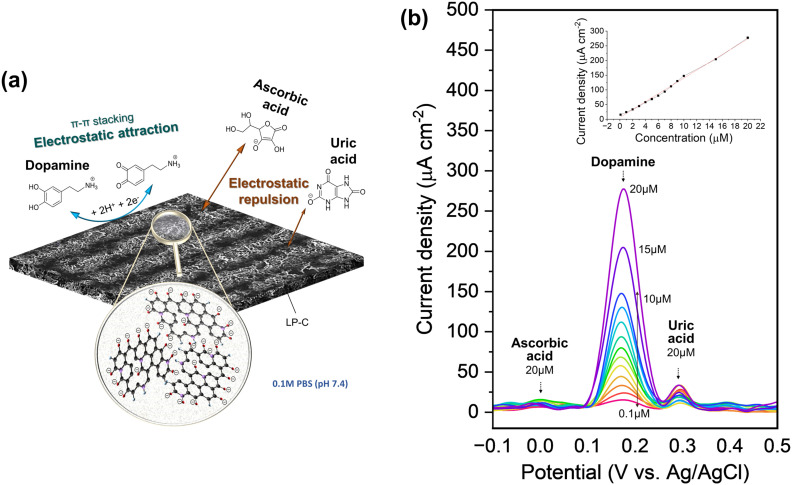
(a) Schematic image of the electrochemical reaction of dopamine (DA), uric acid (UA) and ascorbic acid (AA) with LP-C in pH 7.4 PBS. (b) Differential pulse voltammetry (DPV) detection curves of ternary mixtures (DA, UA and AA), and a (inset) plot of peak current densities as a function of dopamine concentration. A linear detection relationship was obtained (*y* = (13.38005 ± 0.25406)*x* − (6.33028 ± 2.23939)) with a high regression value of 0.996.

At physiological pH 7.4, DA molecules are positively charged (p*K*_b_ 8.87) and UA (p*K*_a_ 5.75) and AA (p*K*_a_ 4.10) molecules are negatively charged.^42^ As can be seen from the material property analysis of LP-C, it has a graphitic oxide surface with oxygenated groups, so it is negatively charged in the electrolyte (pH 7.4 PBS). Therefore, DA can easily access the negatively charged surfaces of LP-C by electrostatic attraction and generate a large electrochemical response (Fig. 5a). In contrast, the LP-C surface has an electrostatic repulsive force toward the negatively charged AA and UA, making them more difficult to detect. In addition, there is a small contribution to the better detection of dopamine. This is because DA has a specific aromatic structure and it further enhances the electronic interaction between the graphitic carbon of LP-C and DA by π–π stacking forces. On the other hand, AA and UA are not aromatic, which is not helpful for the interaction with LP-C.^43–45^ Graphitic carbon surfaces often contain functional groups such as carboxyl (–COOH) or hydroxyl (–OH) groups, which are typically deprotonated at physiological pH of about 7.4 (PBS solution), resulting in a negatively charged surface. However, also modified carbon electrodes have been reported, such as metal, metal oxide, or conductive polymer modified carbon.^46–48^ Due to the modified catalytic properties of these electrodes, uric acid can also be detected as a strong DPV peak.

The excellent selectivity towards DA can be confirmed by differential pulse voltammetry (DPV) using a three-electrode setup (Fig. 5b). As shown in the supercapacitor application, the LP-C has a large capacitive charging current, so its interference is large, making it not sensitive enough to detect redox reactions with standard CV (Fig. S12b†). The differential pulse characteristics in DPV help to reduce interference from non-faradaic currents or background signals, resulting in cleaner and sharper peaks. This allows for better identification and quantification of target redox species, even in complex samples with potential interferences. DA, AA and UA were measured at the same maximum concentration of 20 μM and show clear differences in peak intensity (Fig. 5b). The AA peak is almost undetectable, whereas the intensity of the UA peak varies despite being measured at the same 20 μM UA concentration. A minimum of 0.1 μM DA was measured and the extrapolated LOD (S/N = 3) is 0.5 μM. To ensure the electrode stability for DA sensing, DPV was measured with 10 and 20 μM DA, together with 20 μM UA and 20 μM AA, in a ternary mixture. The 1st, 4th, and 7th days were selected for measurement (Fig. S13†). Each time after measurement, the LP-C electrode was again stored in PBS (pH 7.4) in a refrigerator after being thoroughly rinsed with DI water. The standard deviations of the current density were calculated for 10 μM (5.34 μA cm^−2^; 1.4% deviation) and 20 μM (9.2 μA cm^−2^; 1.3% deviation) DA, respectively. These results indicate high stability of the fabricated electrodes for DA sensing. A comparison of reported carbon electrochemical sensors produced from laser-carbonized biomass-derived precursors (Table S2†).^49,50^ The measured LOD value of this work is the best (0.1 μM) to those in other reports, but shows slightly higher LOD (0.5 μM) from the extrapolated value, but only lower than the study of Ag nanoparticle doped LP-C, which might have an enhanced signal from modified LP-C. In addition, the calibration slope of this work shows the highest sensitivity (13.38 μA μmol^−1^ cm^−2^) for the detection of DA compared to two other studies (0.98 and 4.39 μA μmol^−1^ cm^−2^). However, this comparison is not correct because the other studies tested only with DA and not with ternary mixtures (DA, UA, and AA). Thus, to the best of our knowledge, our LP-C sensor platform has the best selectivity and sensitivity among sustainable (biomass-derived) LP-C DA sensors.

## Conclusion

In this study, we have successfully developed a sustainable-by-design concept to fabricate LP-C electrodes for electrochemical applications, considering (1) resources – using SLS, a biomass waste of the wood pulping process in paper mills, (2) energy consumption – rapid and efficient laser processing, and (3) end-of-life scenario – potentially fully degradable. In contrast, the fabrication of conventional electrochemical carbon electrodes involves several processing steps, from the labor-intensive preparation of the precursor material, to its carbonization and attachment to the current collector. With the concept presented here, LP-C electrodes can be fabricated in a desired pattern and size in a short time with a single laser irradiation step. A key step is the introduction of a polymer interlayer (polystyrene, a global non-recyclable plastic waste) as an adhesive that bonds any substrate (*e.g.* glass, metal, graphite) to the LP-C. With appropriate laser parameters, the polymer interlayer provides mechanical integrity and excellent electrical connection between the porous carbon and the GF current collector. To the best of our knowledge, the supercapacitor and dopamine sensing performance of our high-conductivity LP-C electrodes were the best among biomass-derived LP-C materials reported to date.

The optimized laser-carbonization process produces a microporous material with high specific surface area, which is promising as an electrode material for various electrochemical applications. Depending on the target application (*e.g.* supercapacitors or electrochemical sensors), the electrochemical performance can be further improved by tuning the pore size through optimization of the laser processing parameters and effective material doping. Likewise, due to the tunable fabrication approach, the composition of the LP-C electrode material can be easily tailored to tune the textural and physicochemical properties of the carbonized material. Overall, the electrode design can be extended to different combinations of sustainable precursors and polymer interlayers and deposited on different types of substrates. This new electrode fabrication concept will provide a route to future electrodes, which are safe and sustainable by design.

## Experimental section

### Chemicals

All chemicals were used as received from the vendors: sodium lignosulfonate (Borregaard LignoTech), polystyrene (35 K, Sigma-Aldrich), dopamine hydrochloride (99%, Alfa acer), uric acid (≥99%, Aldrich), l-(+)-ascorbic acid (99+%, Thermo scientific), sodium phosphate dibasic (99%, Thermo scientific), sodium phosphate monobasic (99+%, Thermo scientific), sodium chloride (≥99.5%, Honeywell), sulfuric acid (96%, Roth). Sodium perchlorate (98.0–102.0%, Thermo Scientific).

### Substrate

Graphite foil (GF) was purchased from ProGraphiteShop, with a thickness of 17 μm on removable PET foil (75 μm). The GF has a high temperature resistance (approx. −210 °C to approx. 450 °C under oxidative atmosphere, up to >2500 °C under inert atmosphere or in vacuum). The thermal conductivity of GF is >1400 W mK^−1^ parallel to the foil surface.

### Preparation of the precursor films

Sodium lignosulfonate (SLS) was dissolved in ethylene glycol to obtain a 0.9 g mL^−1^ ink. PS was dissolved in chloroform to obtain a 0.6 g mL^−1^ polymer interlayer solution. All concentrations are given with respect to the volume of the solvent. A drop of the PS solution was applied to the GF and spin-coated at 30 rps for 10 s, which was repeated twice. A drop of the SLS ink was applied to the prepared PS/GF film and the ink was spin-coated at 20 rps for 10 s. Accordingly the film was annealed at 50 °C on a hot plate overnight to obtain the final films with thicknesses of ∼60 μm.

### Laser carbonization

Laser carbonization was conducted with a high-precision laser engraver setup (Speedy 100, Trotec) equipped with a 60 W CO_2_ laser. Focusing was achieved with a 2.5 inch focus lens providing a focal depth of ∼ 3 mm and a focus diameter of 170 μm. The center wavelength of the laser is 10.6 ± 0.03 μm. The scanning speed *ν*, generically given in %, was converted into mm s^−1^. The resulting energy input per distance (or fluence) *F* in J m^−1^ in the cutting mode onto the film is given by *F* = *P*·*ν*′,where *P* is the effective power and *ν*′ is the scanning speed of the laser in s m^−1^.

Laser powers ranging from 34 mW to 920 mW and laser speeds ranging from 2.4 mm s^−1^ to 10.2 mm s^−1^ were used in the screening process for the fabrication of LP-C electrodes, and the laser power of 650 mW and the laser speed of 2.4 mm s^−1^ were used for the optimal conditions for the fabrication of the LP-C electrode.

### Electrochemical measurement

#### Supercapacitor measurement

The electrochemical performance of the LP-C supercapacitors was evaluated using a Gamry instrument (Interface 1000E) and Biologic MPG-2 instrument.

In a 3-electrode cell system, an LP-C electrode was placed as the working electrode in 1 M H_2_SO_4_ with Ag/AgCl as the reference electrode and a Pt mesh as the counter electrode. Cyclic voltammetry (CV) was performed at a scan rate of 10, 50, 100, 200, 500, 1000, 2000, and 5000 mV s^−1^, with a potential window of 0 to 0.8 V. The Nyquist plot of LP-C was obtained at a potential amplitude of 10 mV, a bias voltage of 0 V, and a frequency range of 100 kHz to 10 mHz.

The EDLC performances of LP-C were measured in a symmetrical two-electrode cell by sandwiching a glass fibre (Watman) separator between two LP-C electrodes (0.5 × 0.5 cm^2^) impregnated with 1 M H_2_SO_4_ or 17 M NaClO_4_ electrolyte.

When an EDLC device is connected to a power source, a voltage is applied and positive and negative ions in the electrolyte begin to accumulate on the surfaces of the two electrodes. The positive ions are attracted to the negative electrode and the negative ions are attracted to the positive electrode. An electrical double layer is formed on each electrode surface, which serves as a storage space for electrical charges. The charging process continues until the voltage across the capacitor device reaches its maximum value and it is fully charged. When power is removed from the EDLC and connected to an electronic device that requires electrical power, the stored electrical energy is released to the device. The potential difference between the two electrodes pushes the accumulated cations and anions back into the electrolyte, and the stored electrical energy is released back into the circuit to power the connected electronic device. The discharging process continues until the charge stored in the capacitor is exhausted, at which point the discharged capacitor can be recharged and the process repeated.

Galvanostatic charge and discharge (GCD) tests were performed from 0 to 0.8 V at various current densities of 0.6, 1.2, 2.2 and 12 mA cm^−2^ in 1 M H_2_SO_4_ and from 0 to 1.6 V at 0.2, 0.4 and 1.2 mA cm^−2^ in 17 M NaClO_4_. The capacitance of the devices (*C*_device_, in mF) was derived from the discharge curves of GCD measurements using eqn (2),2
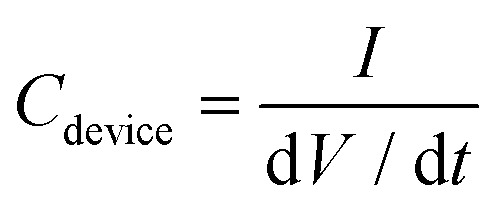
where *I* is the discharge current (in mA), d*V*/d*t* is the slope of the galvanostatic discharge curve after the voltage drop.

The specific areal capacitances (*C*_A_, in mF cm^−2^) of a single electrode can be determined from GCD using eqn (3),3
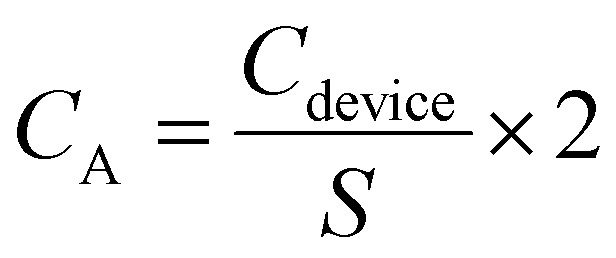
where *S* is the geometric area of the EDLC device (0.25 cm^2^).

The specific areal energy density (μW h cm^−2^) is calculated by integrating the area below discharge curve according to eqn (4),4
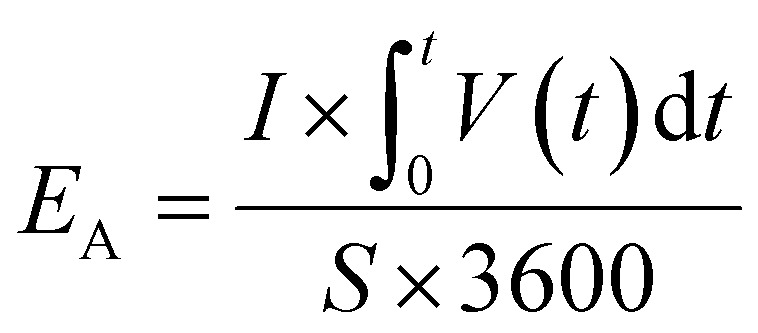
where *V*(*t*) is the device voltage after the ohmic drop, *t* is the discharge time; 
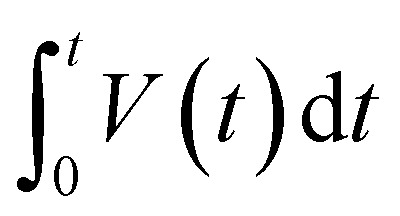
 is the integrated area of the galvanostatic discharging curves.

The specific areal power density (mW cm^−2^) is obtained from the eqn (5),5
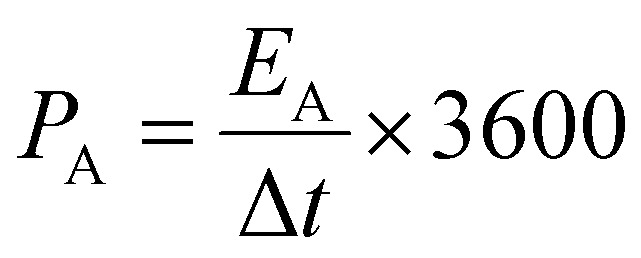
where Δ*t* is the discharge time (s).

Cyclability testing of the LP-C supercapacitor was performed over 20 000 GCD cycles from 0 V to 0.8 V at 2.2 mA cm^−2^ in 1 M H_2_SO_4_ and over 6000k cycles from 0 V to 1.6 V at 2.2 mA cm^−2^ in 17 M NaClO_4_.

The coulombic efficiency (*η*) was calculated from eqn (6),6
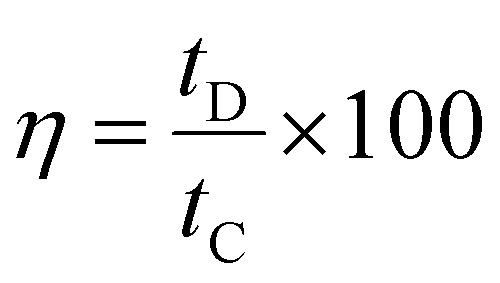
where *t*_D_ is the discharge time and *t*_C_ is the charge time.

#### Galvanostatic intermittent titration technique (GITT)

GITT was tested by cycling the cell at 50 mA g^−1^ for 10 s, followed by an open circuit relaxation period of 30 s. Prior to the GITT test, the device was precycled at 50 mA g^−1^ for ten cycles.

Diffusion coefficients were estimated based on Fick's laws of diffusion, using the following simplified equation:
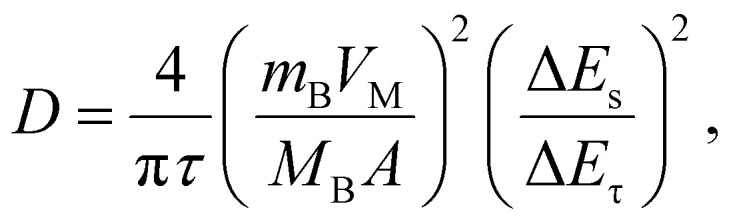
where *τ* represents the duration time of the pulse, *A* is the interface area between the electrode and the electrolyte, *m*_B_, *M*_B_ and *V*_M_ correspond to the mass, molar weight and molar volume of the active material. Δ*E*_s_ (the steady-state voltage variation during a single GITT pulse) and Δ*E*_τ_ (the voltage difference between the constant current pulse and the IR drop) were extracted from each pulse of the typical GITT profile (Fig. S9†).

#### Electrochemical sensing of dopamine (DA), uric acid (UA) and ascorbic acid (AA)

The electrochemical performance of the LP-C electrochemical sensor was evaluated using a Gamry instrument (Interface 1000E, USA). The LP-C electrode was placed in a 3-electrode cell system as a working electrode with 0.1 M PBS (pH 7.4) with Ag/AgCl as a reference electrode and a Pt wire as a counter electrode. CV over a potential range of −0.1 to 0.6 V and a scan rate of 10 and 50 mV s^−1^ with 0.1 M PBS (pH 7.4) in the presence of 0.2 mM DA, 5 mM UA and 5 mM AA. Differential pulse voltammetry (DPV) was also measured with the same concentration of analytes and electrolyte over a potential range of −0.1 to 0.5 V and a scan rate of 10 and 50 mV s^−1^. For the DA concentration study, DPV responses of different DA concentrations in the presence of 20 μM UA and AA were recorded over a potential range of −0.1 to 0.5 V with the following parameters: pulse height of 45 mV, step size of 1 mV, pulse time of 0.03 s, sample period of 0.4 s, and scan rate of 2.5 mV s^−1^.

### Material characterization

#### Scanning electron microscopy (SEM)

SEM measurement were performed on a Zeiss LEO 1550-Gemini system (acceleration voltage: 10 kV). An Oxford Instruments X-MAX 80 mm^2^ detector was used to collect the energy dispersive X-ray (EDX) data.

#### Fourier transform infrared (FTIR)

The FTIR measurement was performed on a Thermo Scientific Nicolet iD7 spectrometer.

#### X-ray diffraction (XRD)

XRD measurement was performed on a Smartlab studio II (Rigaku, Cu K_α_, 0.154 nm) instrument.

#### Raman

Spectra were obtained using a confocal Raman microscope (alpha300R, WITec) equipped with a piezo-scanner (P-500, Physik Instrumente). The laser, *λ* = 532 nm, was focused at the samples through a 100× objective. The laser power at the sample was set to 20 mW.

#### X-ray photoelectron spectroscopy (XPS)

X-ray photoelectron spectroscopy (XPS) measurements were performed using a K-Alpha + XPS spectrometer (ThermoFisher Scientific). The Thermo Avantage software was used for data acquisition and processing. All samples were analyzed using a microfocused, monochromated Al Kα X-ray source (400 μm spot size). The K-Alpha + charge compensation system was used during the analysis, using electrons of 8 eV energy, and low energy argon ions to prevent any localized charge build-up. The spectra were fitted with one or more Voigt profiles (BE uncertainty: ±0.2 eV) and Scofield sensitivity factors were applied for quantification.^51^ Spectra were referenced to the C 1s peak (C–C, C–H) at 285.0 eV binding energy and to sp^2^ species at 284.4 eV, controlled by the known photoelectron peaks of metallic Cu, Ag and Au, respectively.

#### Gas sorption analysis

The specific surface area and pore size distribution were determined by measuring N_2_ and CO_2_ physisorption using a Quantachrome Quadrasorb SI at 77 K for N_2_ and 273 K for CO_2_. Samples were degassed at 120 °C overnight prior to the measurements. The density functional theory (DFT) method was used to evaluate the pore size distribution (PSD) of the materials employing adsorption isotherms.

#### Sheet resistance

The sheet resistivity of LP-C was measured by Ossila 4-point probe (T2001A3) at room temperature.

## Conflicts of interest

The authors declare no conflicts of interest.

## Supplementary Material

NR-016-D4NR00588K-s001
