# Supplementary material for: Sustainable design of high-performance multifunctional carbon electrodes by one-step laser carbonization for supercapacitors and dopamine sensors
Source: Nanoscale. 2024 Apr 9;16(17):8627–38. doi: 10.1039/d4nr00588k (PMC11064777; doi:10.1039/d4nr00588k)
Supplement: NR-016-D4NR00588K-s001 [file NR-016-D4NR00588K-s001.pdf]

Electronic Supplementary Material (ESI) for Nanoscale.
This journal is © The Royal Society of Chemistry 2024

Supporting Information

Sustainable design of high-performance multifunctional carbon electrodes by one-step laser carbonization for supercapacitors and dopamine sensors

Sanghwa Moon,[a] Evgeny Senokos,[a] Vanessa Trouillet,[b] Felix Loeffler*[a] and Volker Strauss*[a]

[a] Max Planck Institute of Colloids and Interfaces, Am Mühlenberg 1, 14476 Potsdam, Germany

[b] Institute for Applied Materials (IAM) and Karlsruhe Nano Micro Facility (KNMFi), Karlsruhe Institute of Technology (KIT), Hermann-von-Helmholtz-Platz 1, 76344 Eggenstein-Leopoldshafen, Germany

* E-mail: felix.loeffler@mpikg.mpg.de, volker.strauss@mpikg.mpg.de

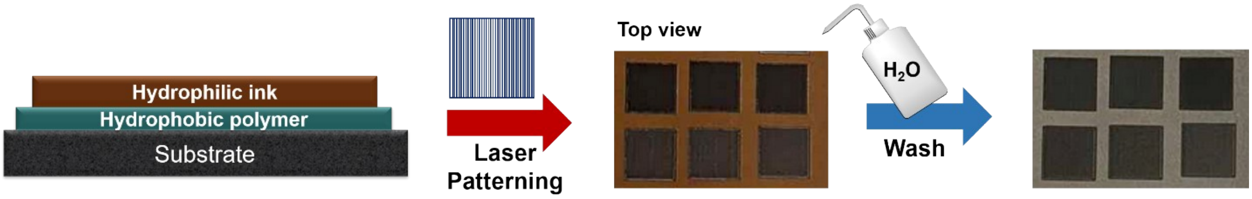

Figure S1. Schematic images showing the process of LP-C fabrication.

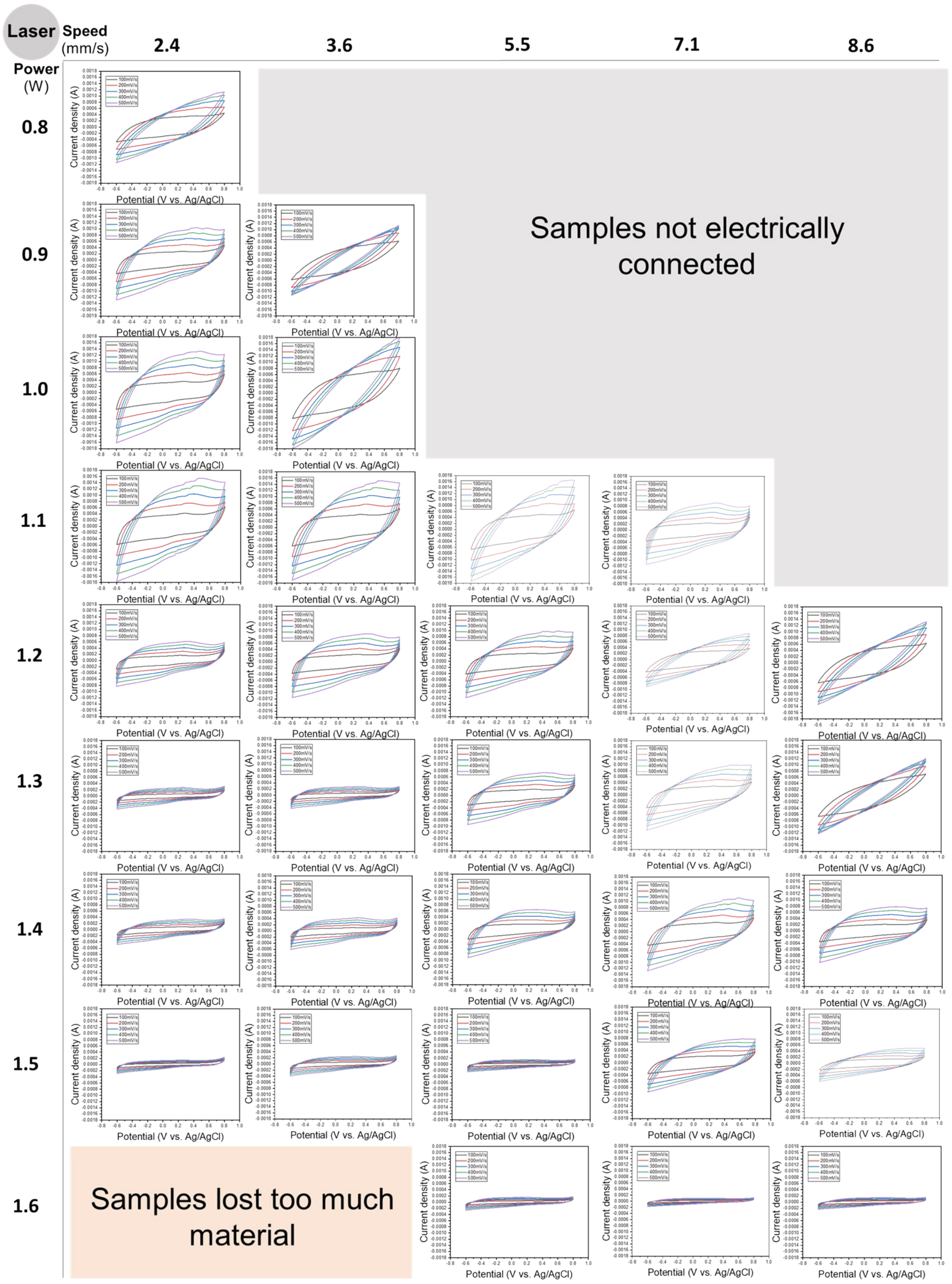

Figure S2. Cyclic voltammetry (CV) curves with varying laser power and speed.

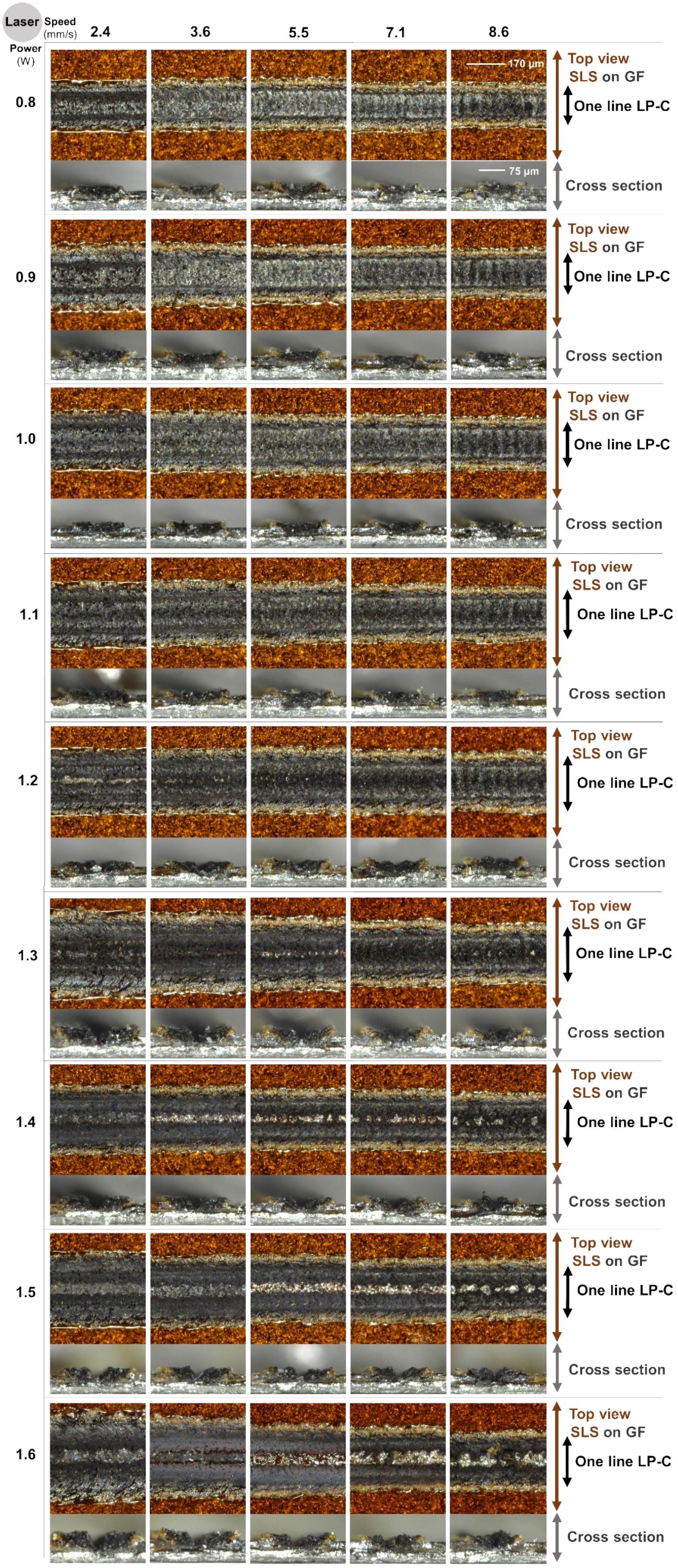

Figure S3. Optical microscope images of a laser-carbonized line obtained in top view and cross-section view with varying laser parameters.

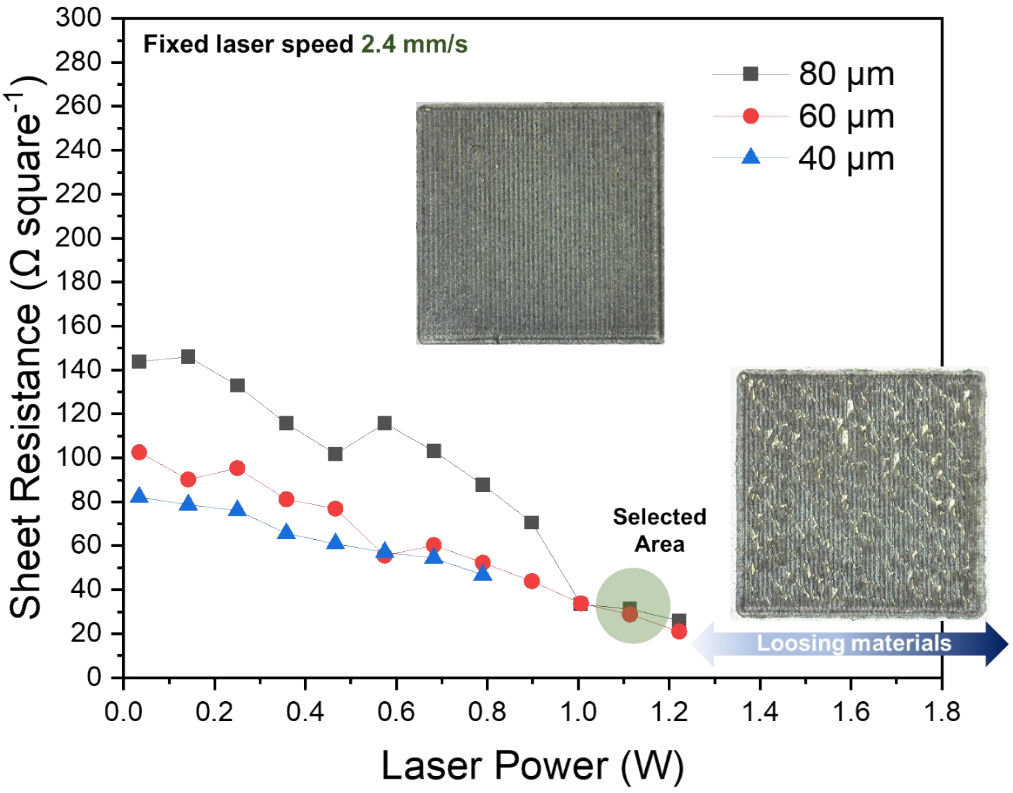

Figure S4. Sheet resistance as a function of sodium lignosulfonate precursor film thickness and laser power at the fixed laser speed (2.4 mm s^{-1}).

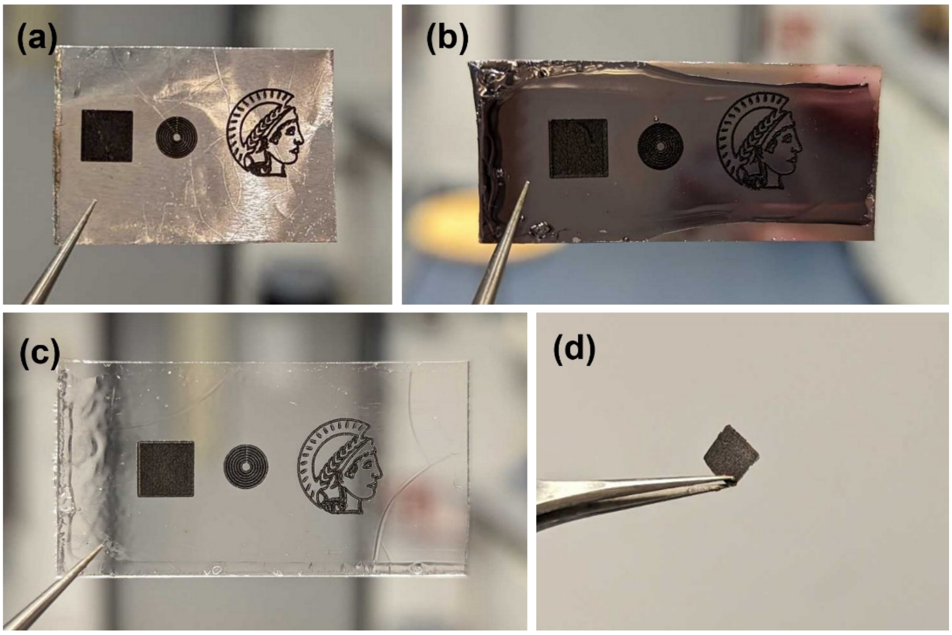

Figure S5. LP-C on (a) Al foil, (b) Si wafer, and (c) glass substrates with different laser patterns; (d) free-standing LP-C.

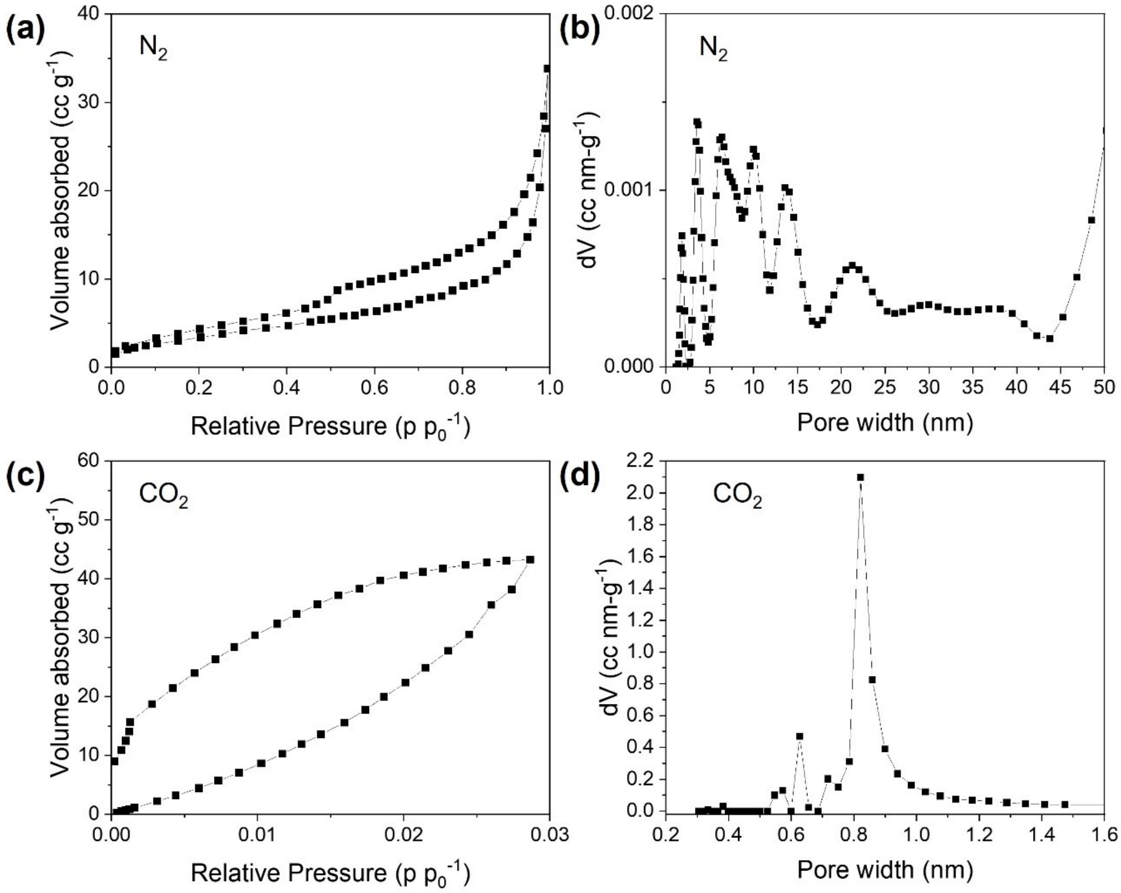

Figure S6. (a) N_2 adsorption–desorption isotherms and (b) pore size distribution of LP-C powder (N_2); (c) CO_2 adsorption–desorption isotherms and (d) pore size distribution of LP-C powder (CO_2).

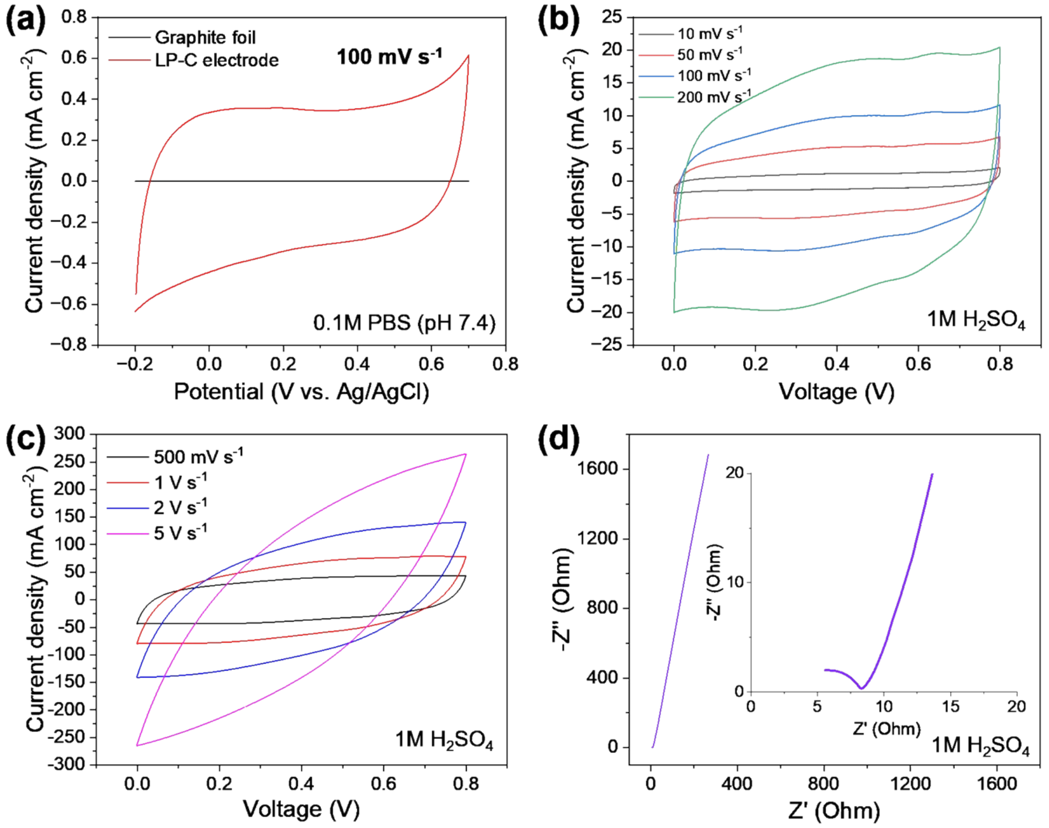

Figure S7. Cyclic voltammetry (CV) curves of LP-C measured with a 3-electrode system (a) in 0.1M PBS (pH 7.4) at 100 mV s^{-1}, (b, c) in 1M H_2SO_4 as a function of voltage scan rate and (d) Nyquist plot.

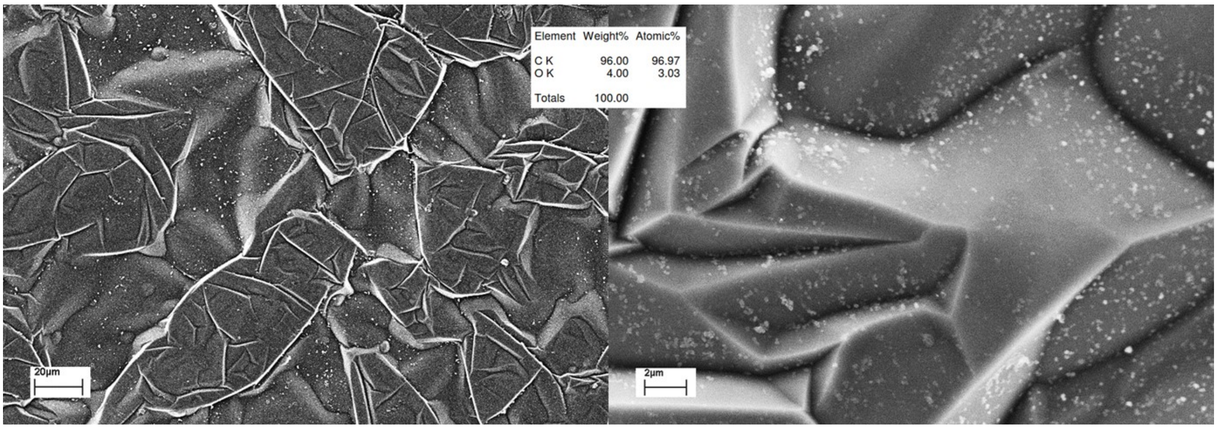

Figure S8. Scanning electron microscope (SEM) images of bare graphite foil.

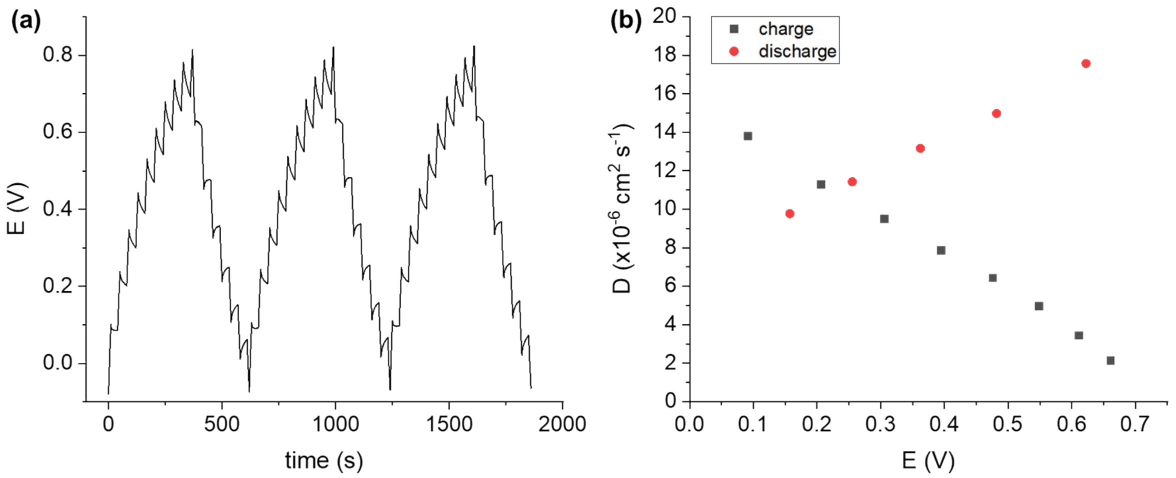

Figure S9. (a) Charge and discharge curves of the galvanostatic intermittent titration technique (GITT) test and (b) diffusion coefficient (D) values as a function of potential tested by cycling a symmetrical cell at 50 mA g^{-1} for 10 sec followed by an open circuit relaxation period of 30 s.

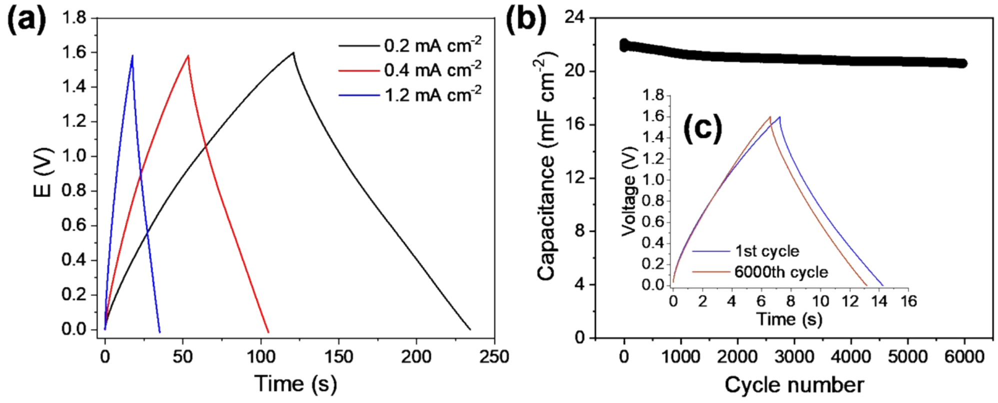

Figure S10. Galvanostatic charge-discharge (GCD) profiles in 17M $NaClO_4$ (a) with different current densities, (b) GCD cyclability test showing the retention of specific areal capacitance at 2.2 mA cm^{-2} and (c) GCD profiles of the 1st and 6000th cycles.

Ref	Precursor	Laser λ (μm)	BET (m^2 g^{-1})	Current collector	Electrolyte	Capacitance (mF cm^{-2})	Energy Density (μWh cm^{-2})	Power Density (mW cm^{-2})	Retention / cycles / Current density (mA cm^{-2})	Current density range (mA cm^{-2})
This work	Sodium lignosulfonate	10.6	13 (N_2), 504 (CO_2)	Graphite foil	H_2SO_4	38.8	1.3 (1.7)	11.4 (14.9)	81% / 20k / 2.2	0.44 - 44
					17M $NaClO_4$	32.8	4.3 (5.8)	16 (21.7)	93% / 6k / 2.2	
35	Kraft Lignin/PVA	10.6	338.3 (N_2)	Copper strip, Silver paint	H_2SO_4/PVA	17	2.6	2.2	99.2% / 12k / 2	0.05 - 2
						6.9	1.3	9.4	-	
36	Lignin/PEO	10.6	-	Copper tape, Silver paint	H_2SO_4/PVA	25.4	3.5	0.8	93% / 4.5k / 0.2	0.1 - 1
37	Kraft Lignin/PEO	10.6	-	Copper tape, Silver paint	H_2SO_4/PVA	2.5	0.3	0.03	91% / 10k / 0.02	0.01 - 0.02
38	Agglomerated Cork	1.06	-	-	H_2SO_4/PVA	1.4	0.1	0.08	106% / 10k / 0.05	0.05 - 0.1
39	Nageia fleuryi leaves	0.346	-	Copper foil, Silver paste	H_2SO_4/PVA	35.3	1.2	0.3	99% / 50k / -	0.005

Table S1. Comparison of the performance and material properties of laser-carbonized supercapacitors using biomass-derived precursors.

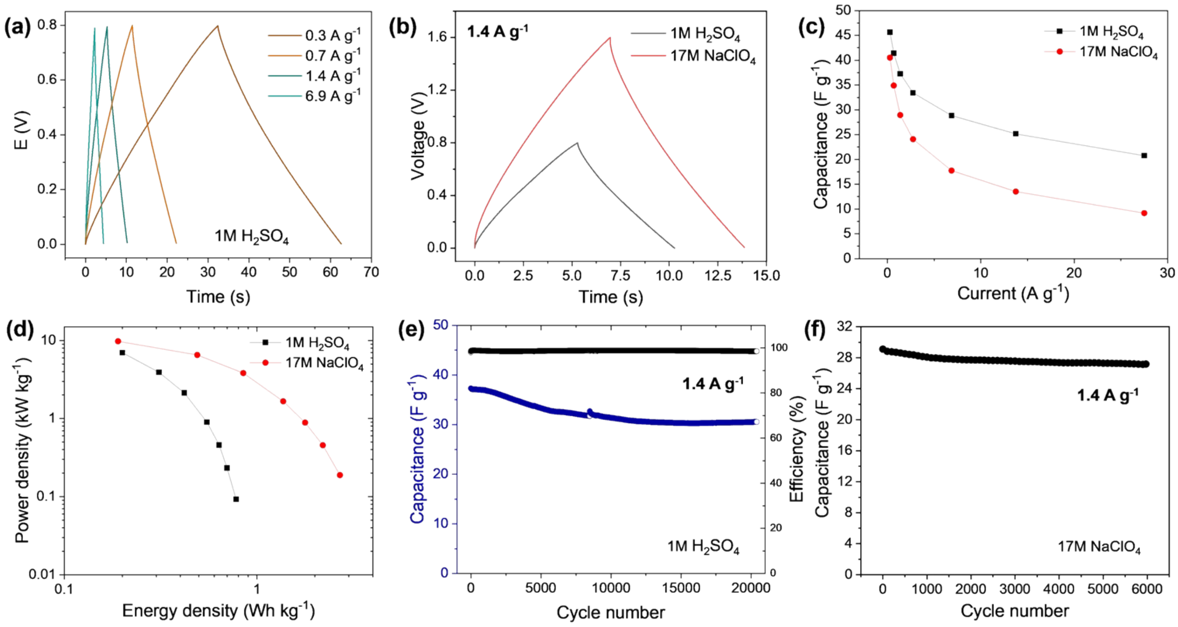

Figure S11. Gravimetric supercapacitor performance of LP-C electrodes using a symmetrical cell: (a) Schematic image of the EDLC charge-discharge process; (b) Galvanostatic charge and discharge (GCD) profiles in 1M H_2SO_4 at different current densities and (c) at a current density of 1.4 A g^{-1} for different electrolytes; (d) Specific gravimetric capacitance as a function of current density; (e) Ragone plot; (f) Long-term cyclability at a current density of 1.4 A g^{-1} showing the retention of specific areal capacitance and coulombic efficiency.

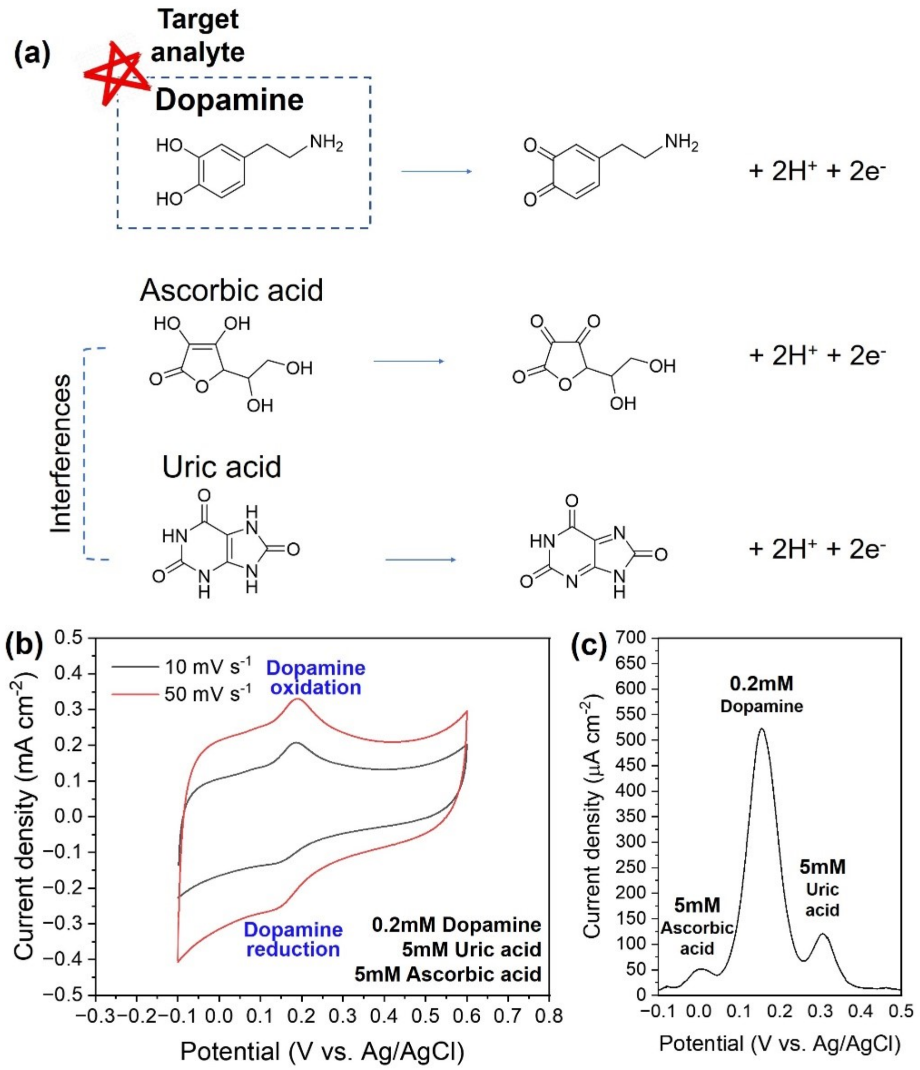

Figure S12. (a) Oxidation process of dopamine (DA), ascorbic acid (AA) and uric acid (UA), (b) cyclic voltammetry (CV) and (c) differential pulse voltammetry (DPV) curves of DA, AA and UA ternary mixtures

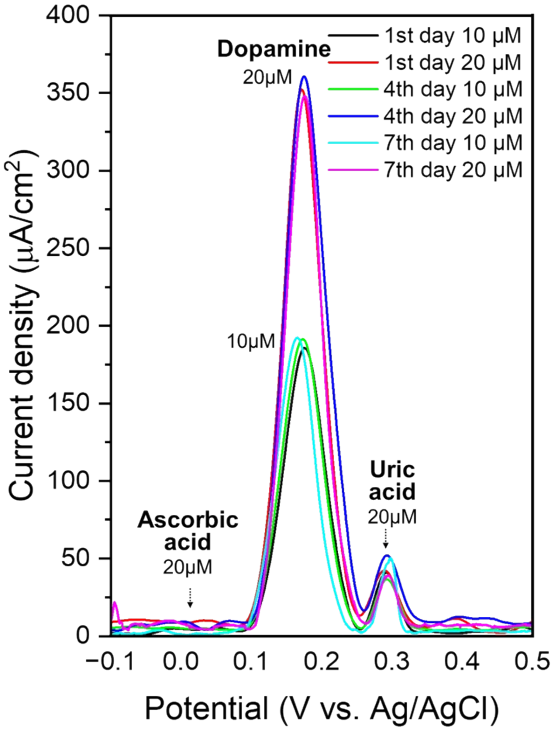

Figure S13. (a) Differential pulse voltammetry (DPV) detection curves of ternary mixtures (dopamine (10 and 20 μM), uric acid (20 μM) and ascorbic acid (20 μM)) on days 1, 4 and 7.

Ref	Precursor	Laser λ	Current collector	Electrolyte	LOD (μM) Measured / Extrapolated	Sensitivity (μA $μM^{-1}$ cm^{-2})	Linear range	R^2	Detection technique
This work	Sodium lignosulfonate	10.6 μm	Graphite foil	0.1M PBS (pH 7.4)	0.1 / 0.5	13.38	0.1 – 20 μM	0.996	CV, DPV
49	Kraft lignin/Cellulose nanofibers	10.6 μm	Glassy carbon	PBS	5 / 3.4	4.39	5 – 40 μM	0.995	CV, DPV
50	Alkaline lignin/Ag NPs/PVA	800 nm	-	0.1M PBS (pH 7.4)	1 / 0.098	0.98	1 – 45 μM	0.99	CV, DPV

Table S2. Comparison of the performance and material properties of laser-carbonized electrochemical dopamine sensors using biomass-derived precursors.
